# Induction of labor versus expectant management of large-for-gestational-age infants in nulliparous women

**DOI:** 10.1371/journal.pone.0180748

**Published:** 2017-07-20

**Authors:** Karolina Moldéus, Yvonne W. Cheng, Anna-Karin Wikström, Olof Stephansson

**Affiliations:** 1 Clinical Epidemiology Unit, Department of Medicine Solna, Karolinska University Hospital and Institutet, Stockholm, Sweden; 2 Department of Obstetrics and Gynecology, Visby Hospital, Visby, Sweden; 3 Department of Surgery, University of California, Davis, United States of America; 4 Department of Obstetrics and Gynecology, California Pacific Medical Center, San Francisco, United States of America; 5 Department of Women’s and Children’s Health, Uppsala University, Uppsala, Sweden; 6 Department of Women’s and Children’s Health, Division of Obstetrics and Gynecology, Karolinska Institutet, Stockholm, Sweden; Stellenbosch University, SOUTH AFRICA

## Abstract

**Background:**

There is no apparent consensus on obstetric management, i.e., induction of labor or expectant management of women with suspected large-for-gestational-age (LGA)-fetuses.

**Methods and findings:**

To further examine the subject, a nationwide population-based cohort study from the Swedish Medical Birth Register in nulliparous non-diabetic women with singleton, vertex LGA (>90^th^ centile) births, 1992–2013, was performed. Delivery of a live-born LGA infant induced at 38 completed weeks of gestation in non-preeclamptic pregnancies, was compared to those of expectant management, with delivery at 39, 40, 41, or 42 completed weeks of gestation and beyond, either by labor induction or via spontaneous labor. Primary outcome was mode of delivery. Secondary outcomes included obstetric anal sphincter injury, 5-minute Apgar<7 and birth injury. Multivariable logistic regression analysis was performed to control for potential confounding. We found that among the 722 women induced at week 38, there was a significantly increased risk of cesarean delivery (aOR = 1.44 95% CI:1.20–1.72), compared to those with expectant management (n = 44 081). There was no significant difference between the groups in regards to risk of instrumental vaginal delivery (aOR = 1.05, 95% CI:0.85–1.30), obstetric anal sphincter injury (aOR = 0.81, 95% CI:0.55–1.19), nor 5-minute Apgar<7 (aOR = 1.06, 95% CI:0.58–1.94) or birth injury (aOR = 0.82, 95% CI:0.49–1.38). Similar comparisons for induction of labor at 39, 40 or 41 weeks compared to expectant management with delivery at a later gestational age, showed increased rates of cesarean delivery for induced women.

**Conclusions:**

In women with LGA infants, induction of labor at 38 weeks gestation is associated with increased risk of cesarean delivery compared to expectant management, with no difference in neonatal morbidity.

## Introduction

The annual incidence rate of fetal macrosomia, often defined as a birthweight above 4500 g, regardless of gestational age, in non-diabetic women, is approximately 3.7%.[1] The term large-for-gestational-age (LGA) is mainly used to define infants with birthweight >90^th^ percentile for gestational age at birth; however, it has been advocated that birthweight >97^th^ percentile (2 standard deviations above the mean) should be used to define LGA as such threshold is associated with higher risk of perinatal morbidity.[2, 3] Regardless of definitions used, the ability to detect a LGA infant is an issue of concern in modern obstetrics because available methods for fetal weight estimation, including ultrasound and clinical measures, are generally imprecise.[3, 4]

The prevalence of macrosomia has increased by 15–25% over the last decades in several developed countries.[3] This increase is largely the result of escalating prepregnancy body-mass-index (BMI), excess gestational weight gain, increased incidence of gestational diabetes mellitus and lowered prevalence of maternal cigarette smoking.[5] Delivery of a macrosomic infant is associated with an increased risk of adverse obstetric outcomes, including instrumental vaginal delivery, cesarean delivery, obstetric anal sphincter injury (OASIS), shoulder dystocia,[1] and postpartum haemorrhage.[6] In addition to shoulder dystocia, perinatal complications include birth asphyxia, and birth trauma such as fractures of the clavicle or humerus and brachial plexus injuries.[6]

Managing pregnancies with a suspected macrosomic fetus is an obstetric dilemma. Whether induction of labor can lower the risk for adverse maternal and infant outcomes remains debatable. Since the fetus gains approximately 280 gram per week on average during the last 3–4 weeks of gestation, induction of labor for suspected LGA-fetuses can be a tempting alternative in an effort to reduce intrauterine weight gain and associated perinatal morbidity.[7] To date, the three published randomized clinical trials studying this topic have reported conflicting results, ranging from induction of labor reduces risk of shoulder dystocia and associated morbidity without increase in cesarean,[8] to no difference in neonatal morbidity but decrease in cesarean,[9] to no difference in morbidity or mode of delivery.[10] A systematic review that included nine observational studies suggested that induction of labor, compared to expectant management, for suspected macrosomia is associated with an increased risk of cesarean delivery without improvement in perinatal outcomes.[7] However, in a recent large observational study, the authors compared nulliparous non-diabetic women induced at 39 weeks of gestation to expectant management, with the assumption of 200-gram intrauterine fetal weight gain per additional week of gestation as an attempt to address continuing intrauterine weight gain with pregnancy prolongation in the expectant management group. This study reported a significantly lowered risk of cesarean delivery in the induced group, and no difference in neonatal outcome, compared to the expectant group.[11]

Currently, there is no apparent consensus on obstetric management of women with suspected LGA-fetuses. Therefore, we conducted a study where we compared mode of delivery, maternal and infant outcomes, of women with LGA infants who underwent induction of labor at 38 completed weeks of gestation or later, to that of expectant management.

## Methods

**Fig 1:**
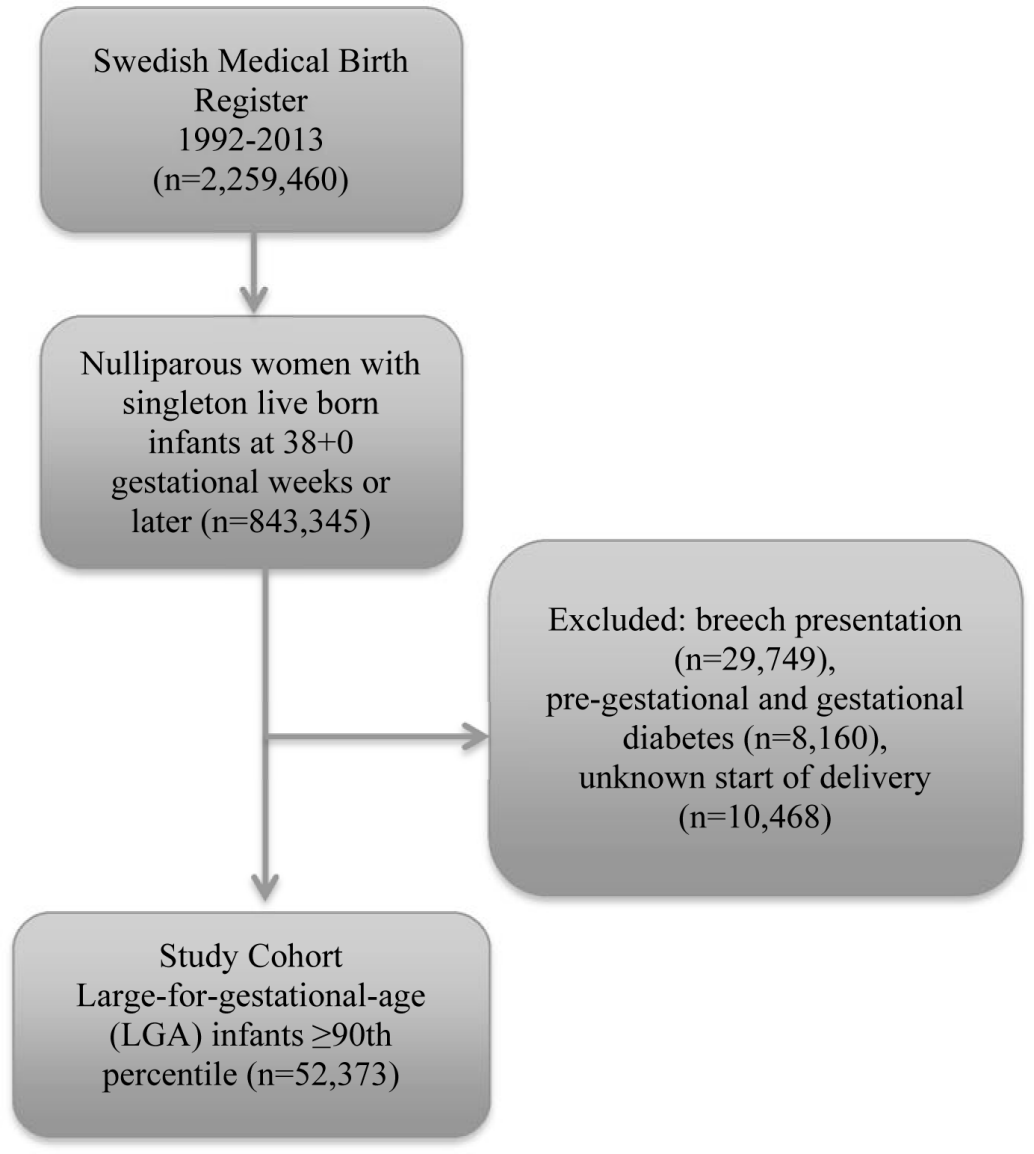
Study cohort flow chart.

This is a population-based cohort study of live singleton births to nulliparous women in Sweden who delivered between 1992–2013 using the Swedish Medical Birth Register. The regional ethical committee at Karolinska Institutet, Stockholm, Sweden approved the study protocol (No. 2008/1182-31/4) and did not require informed consent. We included only live-born LGA infants (above the 90^th^ centile) delivered after induction of labor at 38 weeks gestation (designated as “Induction” group), and those had either spontaneous or induced labor at 39 weeks gestation and beyond (designated as “expectant management” group). We excluded women with breech presentation, pregestational and gestational diabetes mellitus (Fig 1). Women with preeclampsia were excluded in the induction group as we intend to capture women who had induction for suspected fetal macrosomia. Since preeclampsia can develop later in pregnancy and thus considered as a risk of expectant management, women diagnosed with preeclampsia in the expectant management were included for analysis. Women with elective cesarean delivery were not excluded from the expectant group, since suspected macrosomia can be an indication for subsequently scheduled cesarean delivery.

The Birth Register contains data on more than 99% of all births in Sweden, including demographic data, information on reproductive history, and complications during pregnancy, delivery, and the neonatal period.[12] Maternal characteristics including height, weight and smoking are recorded in a standardized manner during a woman’s first visit to antenatal care, which occurs before the 15th week of gestation in more than 95% of the pregnancies[13] and were categorized according to Table 1.

**Table 1:** Maternal and delivery characteristics in singleton births at 38 gestational weeks and onward with birthweight for gestational age above the 90^th^ percentile in primiparous women, Sweden, 1992–2013.

Characteristics	N	%
**Total**	52 373	100.0
**Maternal age (years)**		
13–24	14 268	27.2
25–29	20 140	38.5
30–34	13 088	25.0
35–48	4877	9.3
**Height (cm)**		
130–159	2709	5.6
160–164	8283	17.0
165–169	13 614	28.0
170–200	24 070	49.4
Missing	3697	-
**BMI**		
11.0–18.4	545	1.2
18.5–24.9	24 918	54.5
25.0–29.9	14 462	29.5
≥30	6781	14.8
Missing	6667	-
**Country of birth**		
Nordic	46 827	90.5
Non-Nordic	4947	9.5
Missing	599	-
**Years of Education**		
12 or less	25 015	49.1
More than 12	25 973	50.9
Missing	1385	-
**Smoking during pregnancy**		
Non-smoker	46 004	92.6
Smoker	3658	7.4
Missing	2711	-

Ultrasound for estimation of gestational length has been offered to all pregnant women in Sweden since 1990 and 95% of the women accepts this offer.[13] Since only one routine obstetrical ultrasound is offered nationwide, this is performed in the early second trimester enabling simultaneous fetal anatomic evaluation and confirmation of gestational dating.[14] If ultrasound estimated date of delivery was not available, we estimated gestational length using the first day of the last menstrual period. Information about birthweight was obtained from the standardized pediatric record. Birthweight-for-gestational-age was classified using the mean birthweight for gestational age according to the sex specific Swedish fetal growth curves.[15] Information about onset of labor, fetal presentation and mode of delivery was obtained from the standardized delivery record. Information on preeclampsia, diabetes mellitus and gestational diabetes were obtained from maternal diagnosis at discharge. Information about 5-minutes Apgar scores was obtained from the neonatal record and birth injuries including peripheral nerve injury, fractures, intracranial injury and haemorrhage were obtained from pediatric discharge diagnoses (S1 Text).

The maternal outcomes examined were risks of cesarean delivery, instrumental vaginal delivery, and obstetric anal sphincter injury (OASIS). Apgar score<7 at 5-minutes of birth and composite birth injury were analyzed as neonatal outcome. Multivariable logistic regression model were used to adjust for potential confounding bias. Covariates included in the regression model were: maternal age, height, early-pregnancy BMI, education, cigarette smoking, and maternal country of birth.

**Fig 2:**
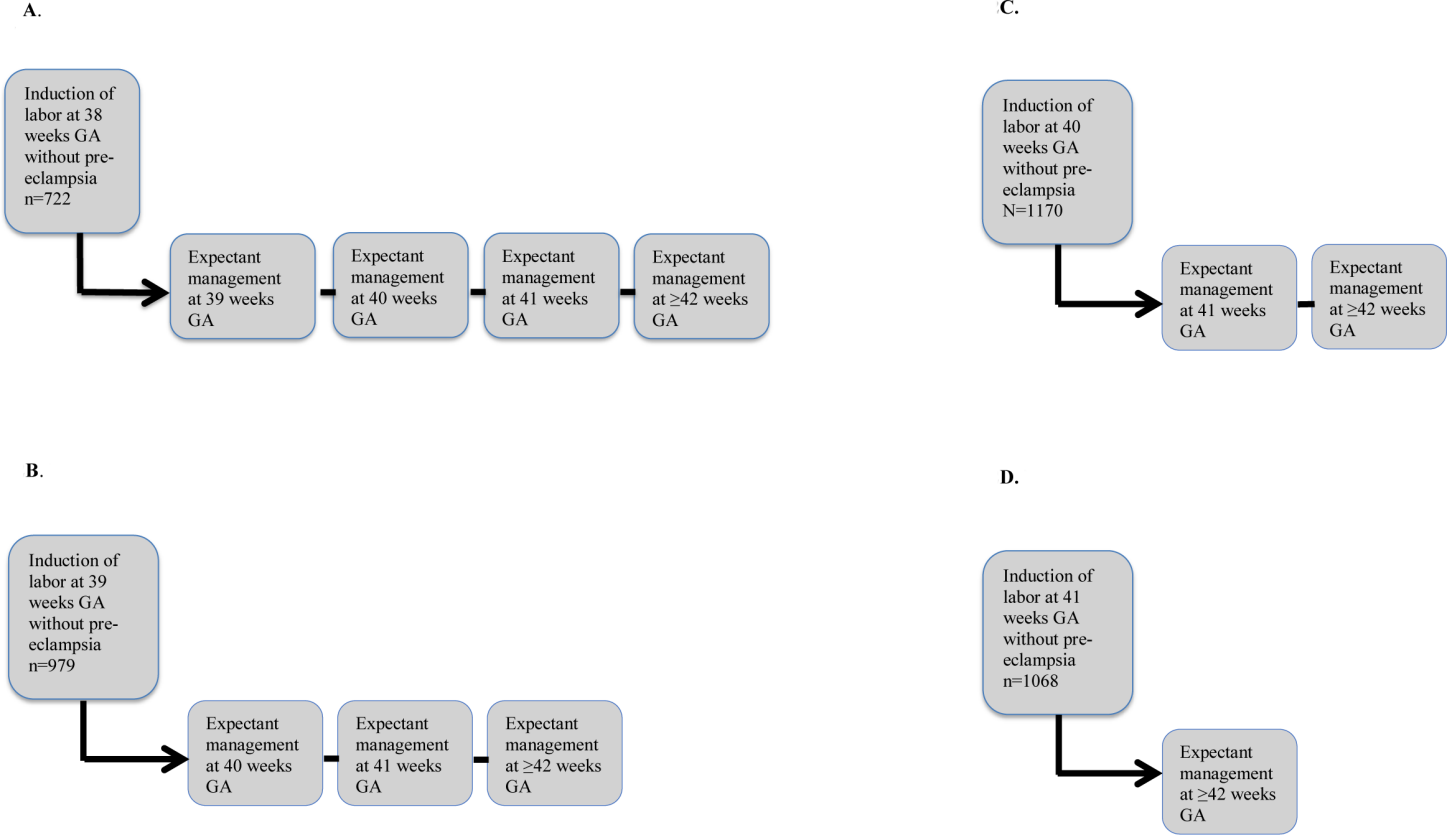
Study comparison groups: Women with LGA infants who underwent induction of labor at one given weeks of gestation were compared to women who similarly had LGA and delivered at a later gestation (at 39, 40, 41, or 42 weeks and beyond), by either spontaneous labor or induction of labor. **Fig 2 A.** Women with LGA infants who underwent induction of labor at 38 weeks compared to women who delivered at 39, 40, 41, or 42 weeks and beyond. **Fig 2 B.** Women with LGA infants who underwent induction of labor at 39 weeks compared to women who delivered at 40, 41, or 42 weeks and beyond. **Fig 2 C.** Women with LGA infants who underwent induction of labor at 40 weeks compared to women who delivered at 41 or 42 weeks and beyond. **Fig 2 D.** Women with LGA infants who underwent induction of labor at 41 weeks compared to women who delivered at 42 weeks and beyond.

We compared the odds of cesarean delivery, instrumental vaginal delivery, OASIS and 5-minutes Apgar<7 in women with LGA infants who underwent induction of labor at 38 weeks gestation to those with expectant management. Because of the different definitions of an LGA-fetus worldwide, we compiled a comparison with a weight span from the 90^th^-97th centile, and greater than the 97th centile, respectively. Each of these weight centile groups delivered at 38 weeks were compared to the corresponding LGA-group delivered at 39, 40, 41 and 42 weeks of gestation as if expectantly managed, accounting for continued intrauterine fetal growth with increasing length of gestation (Fig 2A). Expectant management group was designated as the referent, since it is the most common way of managing pregnancies complicated by LGA in Sweden. The same comparison was performed at gestational week 39, 40 and 41 respectively, compared to expectant management group. (Fig 2B, 2C and 2D). Chi-square test was used for univariate comparison of categorical variables. Crude and adjusted odds ratios with 95% confidence intervals (CI) were calculated by unconditional multivariable logistic regression analysis. Statistical significance was indicated by a p-value of <0.05 and/or 95% CI not containing unity.

## Results

Among the 2 259 460 deliveries recorded in the Birth Register between 1992 and 2013, there were 52 373 women who met the study inclusion/exclusion criteria (Fig 1). Maternal characteristics are presented in Table 1. In gestational week 38 a total of 722 women were induced and 44 081 had expectant management. Of these women, 6066 later underwent induction of labor (13.8%).

We examined the incidence rate of cesarean delivery among women with LGA neonates >90^th^ percentile who underwent induction of labor at 38 weeks (32.7%) compared to those women with LGA neonates who were expectantly managed and delivered at a later (39, 40, 41, or ≥42) weeks of gestation (23.1%, Table 2). The association between remained statistically significantly in the adjusted analysis (aOR 1.44, 95% CI:1.20–1.72; Table 2). Women with LGA who underwent induction of labor at 39, 40 or 41 weeks and beyond had higher odds of cesarean delivery compared to those expectantly managed, and delivered at 40, 41 or 42 weeks and beyond (Table 2). We performed similar comparison of induction versus expectant management and associated risk of cesarean with stratification by LGA categories (90^th^-96.9 centile, and ≥97^th^ centile) (S1 Table). The adjusted odds for cesarean delivery in LGA infants induced at gestational week 38 compared to expectant management were 1.19 (95% CI:0.93–1.54) for the 90^th^-96.9 centile and 1.52 (95% CI:1.18–1.96) for the ≥97^th^ centile, respectively.

**Table 2:** Risk of cesarean delivery associated with induction of labor at a given gestational age, compared to expectant management with delivery at a later gestation, among pregnancies with large-for-gestational-age infants (90^th^ centile and greater).

Week	Induction		Expectant			
	N	Cesarean	N	Cesarean	aOR*	95% CI
38	722	32.7%	44 081	23.1%	1.44	(1.20–1.72)
39	979	32.5%	30 713	25.5%	1.12	(0.96–1.31)
40	1170	41.7%	14 858	31.9%	1.32	(1.15–1.51)
41	1068	48.6%	4449	43.0%	1.10	(0.94–1.28)

*Adjusted for maternal age, height, BMI, education, smoking, country of birth and calendar year.

Women with preeclampsia were excluded from the induction groups. Please see Fig 2 for further information on the expectant group.

The incidence of instrumental vaginal delivery among women with LGA induced at 38 weeks (16.5%) was similar to those who had expectant management (16.2%, aOR 1.05, 95% CI:0.85–1.30, Table 3). The risk of instrumental vaginal delivery among those induced at 39 weeks, 40 weeks, or 41 weeks, compared to their counterparts delivered at a later gestational age were also not statistically significantly different (Table 3). We observed that induction of labor was associated with a lower risk of OASIS among women who underwent induction of labor at 39 and 40 weeks gestation and achieved vaginal delivery: (aOR 0.67, 95% CI:0.47–0.94 and aOR 0.66, 95% CI:0.47–0.93; Table 3). The association between OASIS among women with LGA who underwent induction and delivered vaginally did not reach statistical significance for induction at 38 weeks, nor at 41 weeks GA, compared to expectant management (Table 3).

**Table 3:** Risk of instrumental vaginal delivery and obstetric anal sphincter injury (OASIS) in pregnancies with large for gestational age infants depending on management with induction at 38 to 41 completed gestational weeks or expectant management with labor at next gestational week or later.

**Week**	**Induction**		**Expectant**			
	N	Instrumental VD	N	Instrumental VD	aOR*	95% CI
38	722	16.5%	44 081	16.2%	1.05	(0.85–1.30)
39	979	17.7%	30 713	16.9%	1.13	(0.94–1.35)
40	1170	16.8%	14 858	17.2%	0.97	(0.82–1.16)
41	1068	16.9%	4449	15.9%	1.08	(0.89–1.32)
**Week**	**Induction**		**Expectant**			
	N	OASIS†	N	OASIS†	aOR*	95% CI
38	486	6.6%	33 890	7.2%	0.81	(0.55–1.19)
39	661	6.5%	22 883	7.8%	0.67	(0.47–0.94)
40	682	7.2%	10 111	8.9%	0.66	(0.47–0.93)
41	549	9.8%	2537	10.0%	0.82	(0.58–1.17)

*Adjusted for maternal age, height, BMI, education, smoking, country of birth and calendar year.

†Vaginal births only.

Women with preeclampsia were excluded from the induction groups.

We did not observe a difference in the adjusted odds ratios of 5-minute Apgar score <7 or birth injury among women with LGA who underwent induction of labor at 38, 39, 40, or 41 weeks compared to their counterparts who had expectant management and delivered at a later gestational age (Table 4).

**Table 4:** Risk of five-minute Apgar score less than seven and birth injury among pregnancies with large for gestational age infants depending on management with induction at 38 to 41 completed gestational weeks or expectant management with labor at next gestational week or later.

**Week**	**Induction**		**Expectant**			
	N	Low 5-min Apgar	N	Low 5-min Apgar	aOR*	95% CI
38	719	1.9%	43 930	1.5%	1.06	(0.58–1.94)
39	976	1.9%	30 615	1.6%	1.02	(0.60–1.72)
40	1166	1.7%	14 815	1.7%	0.96	(0.59–1.56)
41	1063	1.7%	4433	2.0%	0.75	(0.42–1.34)
**Week**	**Induction**		**Expectant**			
	N	Birth injury	N	Birth injury	aOR*	95% CI
38	722	2.5%	44 081	3.0%	0.82	(0.49–1.38)
39	979	2.6%	30713	3.1%	1.01	(0.67–1.53)
40	1170	3.3%	14 858	3.3%	1.06	(0.74–1.53)
41	1068	3.8%	4449	3.2%	1.07	(0.70–1.63)

*Adjusted for maternal age, height, BMI, education, smoking, country of birth and calendar year.

Women with preeclampsia were excluded from the induction groups. Apgar-score was not available for all births (n = 154 observations missing).

## Discussion

In this population-based cohort study, there was an overall increased risk of cesarean delivery in women with an LGA infant (≥90^th^ percentile) who had undergone induction of labor at 38 weeks gestation, compared to women who were expectantly managed and delivered at a later gestation by either spontaneous or induced labor. In stratified analysis among women with LGA infants between 90^th^ and 96.9^th^ centile, and of 97^th^ centile and greater, the risk of cesarean remained higher among women induced at 38 weeks gestation compared to expectant management in LGA infants of ≥97th centile. Our findings are in agreement with earlier studies, which report that induction of labor for suspected fetal macrosomia may not be without risks, as induction in this setting may be associated with a higher risk of cesarean delivery.[3, 4, 7, 9, 16] Yet, compared to some of the earlier observational studies, our study utilized a more appropriate study design, which mimics intrauterine physiology by accounting for continued intrauterine weight gain in pregnancies that occurs when pregnancies are expectantly managed with eventual delivery at a later gestational age. Despite differences in study design, our study conclusion was similar.[7, 16]

In contrast, our results differ from those of one study of a U.S. population,[11] which utilized a similar study design as we did to account for intrauterine fetal growth in pregnancies that were expectantly managed. The study found that induction of labor was associated with a lower risk of cesarean delivery compared to expectant management among women with macrosomic infants. There are differences between the U.S. study and our study that could potentially account for the conflicting observation. First, the U.S. study assumed a set amount (200 gram) of intrauterine weight gain per additional week of gestational age among the expectant management group, whereas our study utilized specific birthweight centile to define LGA for gestational age and thus accounting for continual intrauterine weight gain. Secondly, there are different incidence rates of cesarean delivery in the study populations. In the US cohort, the cesarean delivery rates ranged between 35 and 50% compared to the current Swedish cohort with a lower cesarean incidence rate of 20 to 49%. Further, there likely exists differences in management of labor and variation in thresholds for performing cesarean delivery between the two countries. The third factor that could partially account for the observed difference between the two studies is that the prevalence of maternal obesity is much higher in the U.S. compared to Sweden.[17, 18] As obesity is known to be a risk factor for cesarean delivery, it may contribute to variation in labor management and potentially influence clinicians’ threshold for recommending cesarean delivery and other obstetric interventions.[19] Further, it may be that macrosomic fetuses born to women who are obese have a higher likelihood of adipose tissue deposition in such a manner that leads to higher likelihood of labor dystocia and birth injury as well as utero-placental insufficiency, all of which are associated with higher risk of cesarean delivery.[19–21] In our study we adjusted for BMI between the comparison groups, however, we recognize that there could still be unobserved/unmeasured or residual confounding that could not be accounted for simply using statistical models. Our study also differs from the most recent randomized controlled trial by Boulvain et al. (8) They found that induction of labor for suspected LGA fetuses was associated with reduced risk of shoulder dystocia and associated morbidity but no increase in CS. However, their study was based on a different setting and clinical guidelines. Both nulliparous and parous women were included, with a relatively high incidence of CS (28% in the induction group and 32% in the expectant group). Induction of labor in the Boulvain study was performed between 37+0–38+6 weeks of gestation and the birth weights were 3831 g in the induction group versus 4118 g in the expectant group. One limitation of our study is the lack of estimated birth weight. The Boulvain study included approximately 400 women in each group and hence had reduced power to investigate these outcomes in greater detail, as we were able to do in the present study. Therefore, our observational study adds valuable knowledge to this field of research.

Interestingly we note that in our study cohort, 13,8% of the women who were expectantly managed with eventual delivery at a later gestational age subsequently underwent induction of labor, either due to medical or obstetric indications. In such a scenario, these women who were expectantly managed eventually became exposed to the theoretical risks associated with labor induction but at a later gestational age with higher birthweight and potentially reduced placental function than if delivery were to have occurred earlier. Induction of labor has traditionally been perceived to be associated with increased risk for cesarean delivery.[22] Yet, this notion largely steamed from historical studies that compared women who had induction to women who had spontaneous labor at similar gestational ages as oppose to actual clinical scenario where women would either undergo induction of labor at a given gestational age or they would be expectantly managed and be delivered at a later gestational age. A recent systematic review suggests that elective induction of term pregnancies with intact membranes is associated with reduced risk of cesarean delivery.[23] Two U.S. cohort studies did not observe an increase of cesarean delivery after induction of labor compared to expectant management, regardless of cervical status.[24, 25] However, neither of these studies specifically examined the association between induction of labor and mode of delivery among pregnancies complicated by suspected fetal macrosomia or LGA infants.

We observed a lower rate of OASIS among women who undergone induction at 39 and 40 weeks gestations compared to their counterparts that were expectantly managed. This is in contrast to the randomized controlled trial by Boulvain where no significant difference was found between the induction and expectant groups.[8] We attribute this potential protective association between induction and OASIS at 39 and 40 weeks gestations to the absolute lower birthweights of the fetuses in the induction group compared to the expectant group and delivered at a later gestational age despite these were all LGA infants. As severe OASIS can be associated with both short term and long term maternal morbidity, including fecal and/or urinary incontinence as well as dyspareunia,[26, 27] it is essential to recognize that the mother´s concern about a possible sphincter injury could potentially outweigh the potential risk of morbidity associated with cesarean delivery. Thus, the decision of whether to recommend induction of labor versus expectant management, in the presence of suspected LGA, should be thoughtfully weighed and the mother’s preferences incorporated in the detailed counseling in order to truly balance potential risks and benefits of management options.

The main strength of this investigation is the population-based study design, where appropriate groups are compared using the LGA-definition. We were able to examine risks of instrumental vaginal deliveries and OASIS associated with induction, which are important outcomes in the obstetric population. One limitation of this study is that we did not use birthweight estimated by ultrasound in late pregnancy as the exposure of interest. Since the decision to undergo induction or expectant management would be made prior to the precise knowledge of birthweight, ideally, we should assign treatment group based on estimated fetal weight; however, this information was not available for all parturient and thus actual birthweight was utilized. Another limitation is that we did not have information regarding the precise indications of or the methods used for labor induction in this study. A limitation is also the lack of information about Bishop score for the induction of labor. Since we only included nulliparous women, the group should be rather homogenous with a generally low Bishop score. The cervical status can however affect the clinician’s decision of inducing the labor or not, and consequently affect our results.

We recognize that women who undergone induction of labor might be at higher risk of cesarean not due to actual induction but the underlying pathophysiology of induction indication aside from fetal macrosomia. However, we were able to identify and exclude women who undergone induction of labor for preeclampsia, pre-gestational or gestational diabetes mellitus, which likely accounted for a majority of women who undergo induction of labor at term. Finally, by using an early second, instead of a first trimester ultrasonography to estimated gestational age, gave our results less precision, since the accuracy in early second trimester dating is +- 7–10 days compared with +- 5–7 days in the first trimester.[28]

In summary, our study demonstrates a higher risk of cesarean delivery after induction of labor in LGA fetuses, compared to expectant management. We observed that induction of labor decreased the rate of OASIS among those induced at 39 and 40 weeks compared to expectant management. Thus, the question of induction of labor versus expectant management is a trade-off between cesarean delivery and OASIS, each with short-term and long-term morbidities. There is however, a great need for larger randomized clinical trials to verify these findings and to develop better methods of measuring excessive fetal growth in term pregnancies. Until such data becomes available, our study suggests that the decision regarding induction of labor, for this group of fetuses suspected to be LGA, should balance the risks associated with cesarean delivery versus OASIS while incorporating maternal preferences. Induction of labor should not be recommended in general.

## Supporting information

S1 Text Discharge ICD-codes used for classification. (DOC)

S1 Table Risk of cesarean delivery in pregnancies with infants between 90–96.9 and ≥97 percentiles in weight for gestational age depending on management with induction at 38 to 41 completed gestational weeks or expectant management with labor at next gestational week or later. (DOCX)
