# Supplementary material for: Induction of labor versus expectant management of large-for-gestational-age infants in nulliparous women
Source: PLoS One. 2017 Jul 20;12(7):e0180748. doi: 10.1371/journal.pone.0180748 (PMC5519027; doi:10.1371/journal.pone.0180748)
Supplement: S1 Text — (DOC) [file pone.0180748.s001.doc]

**S1 Text.** Discharge codes used for classification

Diagnoses for pregestational and gestational diabetes and preeclampsia using 10^th^ revision of the International Statistical Classification of Diseases [ICD-10] codes: O14, O15 and O24; and the 9^th^ revision [ICD-9] codes: 642E-642H, 648A and 648W.

Diagnoses for birth injuries including peripheral nerve injury, fractures, intracranial injury and haemorrhage (ICD-10 codes: P10, P13 and P14; and ICD-9 codes: 767A, 767C, 767D, 767F, 767G, 767H and 767X).
