# Supplementary material for: Induction of labor versus expectant management of large-for-gestational-age infants in nulliparous women
Source: PLoS One. 2017 Jul 20;12(7):e0180748. doi: 10.1371/journal.pone.0180748 (PMC5519027; doi:10.1371/journal.pone.0180748)
Supplement: S1 Table — (DOCX) [file pone.0180748.s002.docx]

**S1 Table.** Risk of cesarean delivery in pregnancies with infants between 90-96.9 and **≥**97 percentiles in weight for gestational age depending on management with induction at 38 to 41 completed gestational weeks or expectant management with labor at next gestational week or later

|  | **90-96.9 percentile** | | | |  |  |
| --- | --- | --- | --- | --- | --- | --- |
| **Week** | **Induction** | | **Expectant** | |  |  |
|  | N | Cesarean | N | Cesarean | aOR* | 95% CI |
|  |  |  |  |  |  |  |
| 38 | 400 | 26.0 % | 29 597 | 19.5 % | 1.19 | (0.93-1.54) |
|  |  |  |  |  |  |  |
| 39 | 554 | 26.5 % | 20 716 | 21.7 % | 1.01 | (0.81-1.26) |
|  |  |  |  |  |  |  |
| 40 | 710 | 37.5 % | 10 055 | 28.0 % | 1.27 | (1.06-1.53) |
|  |  |  |  |  |  |  |
| 41 | 675 | 45.0 % | 3002 | 38.0 % | 1.16 | (0.95-1.41) |
|  |  |  |  |  |  |  |
|  | **≥97 percentile** | | | |  |  |
| **Week** | **Induction** | | **Expectant** | |  |  |
|  | N | Cesarean | N | Cesarean | aOR* | 95% CI |
|  |  |  |  |  |  |  |
| 38 | 322 | 41.0 % | 14 484 | 30.5 % | 1.52 | (1.18-1.96) |
|  |  |  |  |  |  |  |
| 39 | 425 | 40.2 % | 9997 | 33.4 % | 1.10 | (0.87-1.38) |
|  |  |  |  |  |  |  |
| 40 | 460 | 48.3 % | 4803 | 40.2 % | 1.25 | (1.01-1.56) |
|  |  |  |  |  |  |  |
| 41 | 393 | 54.7 % | 1447 | 53.3 % | 0.92 | (0.71-1.20) |
|  |  |  |  |  |  |  |

*Adjusted for maternal age, height, BMI, education, smoking, country of birth and calendar year.

Women with preeclampsia were excluded from the induction groups.
